# Neurobiology of Maternal Stress: Role of Social Rank and Central Oxytocin in Hypothalamic–Pituitary Adrenal Axis Modulation

**DOI:** 10.3389/fpsyt.2015.00100

**Published:** 2015-07-07

**Authors:** Jeremy D. Coplan, Asif Karim, Prakash Chandra, Garleen St. Germain, Chadi G. Abdallah, Margaret Altemus

**Affiliations:** ^1^Department of Psychiatry and Behavioral Sciences, Nonhuman Primate Facility, State University of New York Downstate Medical Center, Brooklyn, NY, USA; ^2^Department of Psychiatry and Behavioral Sciences, Kansas University Medical Center, Kansas City, KS, USA; ^3^Department of Psychiatry, Yale School of Medicine, New Haven, CT, USA; ^4^Department of Psychiatry, Weill Cornell Medical College, New York, NY, USA

**Keywords:** early-life stress, variable foraging demand, subordinate stress, cortisol, attachment security, oxytocin

## Abstract

**Background:**

Chronic stress may conceivably require plasticity of maternal physiology and behavior to cope with the conflicting primary demands of infant rearing and foraging for food. In addition, social rank may play a pivotal role in mandating divergent homeostatic adaptations in cohesive social groups. We examined cerebrospinal fluid (CSF) oxytocin (OT) levels and hypothalamic–pituitary adrenal (HPA) axis regulation in the context of maternal social stress and assessed the contribution of social rank to dyadic distance as reflective of distraction from normative maternal–infant interaction.

**Methods:**

Twelve socially housed mother–infant bonnet macaque dyads were studied after variable foraging demand (VFD) exposure compared to 11 unstressed dyads. Dyadic distance was determined by behavioral observation. Social ranking was performed blindly by two observers. Post-VFD maternal plasma cortisol and CSF OT were compared to corresponding measures in non-VFD-exposed mothers.

**Results:**

High-social rank was associated with increased dyadic distance only in VFD-exposed dyads and not in control dyads. In mothers unexposed to VFD, social rank was not related to maternal cortisol levels, whereas VFD-exposed dominant versus subordinate mothers exhibited increased plasma cortisol. Maternal CSF OT directly predicted maternal cortisol only in VFD-exposed mothers. CSF OT was higher in dominant versus subordinate mothers. VFD-exposed mothers with “high” cortisol specifically exhibited CSF OT elevations in comparison to control groups.

**Conclusion:**

Pairing of maternal social rank to dyadic distance in VFD presumably reduces maternal contingent responsivity, with ensuing long-term sequelae. VFD-exposure dichotomizes maternal HPA-axis response as a function of social rank with relatively reduced cortisol in subordinates. OT may serve as a homeostatic buffer during maternal stress exposure.

## Introduction

Exposure to early-life stress increases susceptibility to the development of, among other conditions, mood and anxiety disorders in humans (1, 2). Because of a shared primate ancestry with humans, as well as similarity to humans by virtue of complexity in social structure, affective expression, genetic similarity, and maternal–infant bonding patterns (3), non-human primates provide a cogent model to facilitate understanding of the neurobiological underpinnings of certain forms of human psychiatric disorders as related to early-life stress exposure (4, 5). Whereas early-life stress in humans is quite variable in its forms and entails multiple factors with complex interactions (6), early-life stress in animal models can be experimentally controlled by narrowing the number of relevant variables and minimizing exposure to stress thereafter (7). In socially housed non-human primate mothers, the search for food is a primary ecological constraint that affects individual and social behavior and impacts on infant rearing patterns (7). Variable foraging demand (VFD) is an experimental model of early-life stress that manipulates maternal accessibility to food in a social environment, putatively disrupting maternal–infant dyadic attachment and creating unpredictable rearing conditions for non-human primate infants (8). We have previously hypothesized that the VFD-stressor interferes with the normative repertoire of maternal rearing patterns including a process termed as maternal “contingent responsivity” (8). Following the VFD form of early-life stress, grown bonnet macaques’ exhibit persistent biobehavioral characteristics that bear resemblance to those observed in human mood and anxiety disorders, including persistent abnormalities of mood-regulating neurochemical systems (9–11) and long-term alterations of brain structures, which are key components to affective neurocircuitries (12–15).

Despite a large body of literature focusing on sequelae in offspring (16), comparatively fewer reports focus on maternal vulnerability and its attendant neurobiology (17, 18). Here, we focus on the plasticity of maternal homeostatic responsivity under conditions of socioecological stress (19) in relation to social rank and infant attachment, as reflected by dyadic distance. We also examined the role of cerebrospinal fluid oxytocin (CSF OT) in the context of maternal social stress, social rank, and HPA-axis regulation. The role of social rank in determining patterns of maternal care in social groups has long been acknowledged (20, 21). However, the role of social rank, particularly among males, varies as a function of a variety of stressors (22–24) and, evidence suggests, becomes increasingly “relevant” under conditions of high and unpredictable resource demand (25). As our first dependent measure, we used maternal–infant distance, a measure of dyadic proximity, as a putative indicator of attachment patterns that may be adaptive to socioecological demand but are distracting in lieu of normative infant nurturance (26). The use of dyadic distance has precedent in the “Hinde Index,” proposed by Hinde and Atkinson, which represents the contribution of each partner of the maternal–infant dyad to the maintenance of proximity between them (27). Although the index is not significantly related to specific behavioral measures (28), we hypothesized that the extent to which social rank predicted dyadic distance would provide a reflection of “rank relevance” and would necessitate, by inference, compromise of the normative process of contingent responsivity. Our hypothesis would be supported by the observation that the enhanced relevance of social rank to dyadic distance would be accompanied by parallel effects on neuroendocrine responsivity, as reflected by plasma cortisol and CSF OT concentrations.

In a meta-analysis, Abbott and colleagues reported that non-human primate groups with stable dominance hierarchies exhibited, depending on a range of factors, variability in the relationship between social rank and plasma cortisol levels (21). Sapolsky reported that higher ranked male savannah baboons in stable hierarchies normally exhibit lower levels of baseline plasma cortisol levels (22, 23), but higher levels of cortisol are exhibited by socially dominant baboons who are less sophisticated in interactions with other males or who display less affiliative behavior (24). In contrast, higher levels of cortisol may also be exhibited by socially subordinate baboons undergoing social stress or experiencing low levels of social support (24). We hypothesized that the social instability engendered by maternal VFD exposure would result in a relative increase in plasma cortisol in socially dominant versus subordinate subjects. The basis for this hypothesis of divergent HPA-axis response is that the VFD stressor would create instability for dominants, who then exhibit HPA-axis activation, whereas subordinates, in the face of a perceived shortage of food with reduced access, may reduce cortisol to restrict stress-induced catabolism (29) of valuable caloric stores required for maternal care.

We also analyzed the role of cerebrospinal fluid (CSF) oxytocin (OT) in the context of social rank and HPA-axis regulation under normative states in comparison to conditions of maternal socioecological stress. Macaque species, such as bonnets, that display high levels of affiliative behaviors, exhibit elevations of CSF OT in comparison to less gregarious species, such as pigtail macaques (30) suggesting an important role in affiliation. OT, as mentioned, plays a significant role in maternal–infant bonding, as demonstrated in a study where infusion of an OT receptor antagonist in the medial prefrontal cortex impaired maternal care behaviors and enhanced maternal aggression in postpartum females (31).

Evidence has emerged that social subordinance, a high “threat state” in rhesus macaques, is associated with reduced serum OT (32). In addition, central expression of OT receptors has been demonstrated to facilitate consolidation of dominance hierarchies (33). Recent evidence suggests that OT may facilitate social interaction by reducing a state of vigilance toward potential social threats (34). Peripheral measures of OT, however, may not be reflective of CNS levels, enhancing the value of cisternal CSF measures (35). Taken together, we hypothesized that central measures of OT would be lower in subordinate versus dominant bonnet macaque mothers. However, it remained unclear whether the CSF OT relationship to social rank would either be influenced by, or impervious to, experimentally induced maternal stress induced by VFD exposure.

Another mechanism posited for OTs pro-social effects is its capacity to inhibit the HPA-axis (36, 37). A recent study investigated the role of parent–child interaction in influencing OT/HPA-axis interactions. Exogenous administration of OT to caregivers experiencing high parent–infant “synchrony” led to an increase of infant HPA reactivity in response to the experimental stress paradigm. In contrast, exogenous administration of OT to caregivers experiencing low parent–infant synchrony led to a decrease in infant HPA reactivity to stress (38). We therefore wished to examine the influence of OT on the HPA-axis under basal conditions in comparison to maternal VFD exposure. Our hypothesis was that under conditions of maternal VFD exposure and heightened social rank relevance, chronic cortisol increases among dominant VFD-exposed females may necessitate relative elevations of OT to contain HPA-axis activation. Relative HPA-axis suppression in VFD-exposed subordinate females would not necessitate OT overdrive with an absence of OT elevations. This hypothesis is partially consistent with recent work where physically abused female, but not male, children, exhibited increased urinary OT but reduced salivary cortisol in comparison to non-maltreated controls (39). In the latter, cortisol reductions may represent a persistent HPA-axis alteration, consistent with our findings following VFD rearing (4) and is also hypothesized to occur in socially subordinate mothers exposed to VFD stress.

## Materials and Methods

### Subjects

Twenty-three mother–infant bonnet macaques (Macaca Radiata) dyads, which have, in part, been previously reported upon (40), served as subjects. Dyadic distance and CSF OT concentrations have not been reported previously. Moreover, maternal VFD versus non-VFD exposure comparisons have not been reported. Dyads were socially housed and divided into four pens of approximately five to seven dyads. By virtue of the harem design of the VFD procedure (7), offspring of mothers within a given pen were sired by a single male, therefore, making all infants within a pen *de facto* half siblings. However, it was required that mothers not be related. Twelve mother–infant dyads housed in two pens were exposed to VFD conditions and served as the experimental group, whereas 11 mother–infant dyads housed in the two remaining pens were not exposed to VFD conditions (i.e., fed *ad libitum*) and served as the control group. The VFD group contained seven male and five female infants, while the non-VFD group contained two male and nine female infants. Maternal parity data were not available. Details of statistical comparisons of maternal and infant characteristics are provided in the results section. The study was approved by the Institutional Animal Care and Use Committee of SUNY Downstate Medical Center.

#### Housing

Subjects were housed in four pens approximately 2 m × 4 m × 2 m in dimension with the 4-m representing the depth of the pen with five to seven animals per pen. VFD dyads were housed separately from non-VFD subjects. Perches were available at two levels within each pen. Water was available at all times from an automated watering system with animal-activated spigots. All pen walls were opaque. Two large one-way hinged glass windows were located at the front of the pen for investigator observations.

### VFD procedures

After infants reached at least 2 months of age, each pen containing five to seven dyads each was subjected to standard VFD procedures, which involved eight alternating 2-week blocks (total 16 weeks) in which food was either easily accessible (low foraging demand: LFD) or more difficult to obtain (high foraging demand: HFD). The difficulty in obtaining food was varied through the use of the “foraging cart,” a device in which food can be hidden in differing amounts, with apertures on the side of the cart through which the mother searches for food (7).

### Behavioral observations

#### Social Rank

Each mother was assessed for social rank rated from most dominant (accorded a score of 1) to most subordinate (accorded a score of 5–7 depending on the pen) during the final LFD phase of the 16-week VFD cycle (VFD ends on a LFD cycle) to approximate *ad libitum* foraging conditions being utilized in both the VFD and non-VFD groups. Maternal social rank was determined by two blinded observers assessing each maternal subject’s behavioral response in agonistic encounters until agonistic encounters with every other subject in the pen had been tabulated. Dominance was attributed to a specific maternal subject when they prevailed in an agonistic encounter, whereas the other subject was denoted as a subordinate when they withdrew from an agonistic encounter. The observers were trained to observe for subordinate hierarchical behavior, including *Displacement*: a subordinate flees at the approach of another in the absence of overt threat or aggression; *Lip smack/crouch/present*: a subject (subordinate) evinces rapid opening–closing of lips with repeated tongue extrusion or suddenly lowers its chest to the floor and/or raises its rump in the direction of a dominant. Dominant hierarchical behavior includes: *Threat*: a subject shows an aggressive facial expression with ears retracted, mouth open, and teeth exposed, directed at another; *Aggressive Chase*: a subject rapidly chases and displaces another, concluding without physical aggression; *Mounting*: subject mounts another subject without sexual intent. An overall social rank was tabulated by each observer for each subject. Not infrequently, in the instance of mid-ranking subjects, rank ambiguity may be present. In these situations, the observer was required to observe an additional three agonistic encounters between the subjects in which rank was disputed. An independent assessment of mother’s social rank status was provided by each of the two experienced, blinded observers with a kappa score >0.95 (one disagreement by one rank for one mid-ranking subject in one pen). Social rank was thus determined in the final LFD phase of the VFD paradigm and at a corresponding time point in the control subjects.

#### Maternal-Infant Proximity (Dyadic Distance)

During daily behavioral sessions, each mother–infant dyad was observed for 20 s in a random sequence on five separate occasions. Dyadic distance was scored during each observation on a scale of 1–5. A score of 1 was given when mother and infant were in direct physical “ventral–ventral” contact. A score of 5 was given when the dyad was at a maximum distance in the pen, as ­permitted by the pen dimensions. A score of 2 was awarded when the mother and the infant were <1 m apart. A score of 3 was awarded if maternal–infant distance was >1 m. A score of 4 was awarded when maternal–infant distance was in between a 3 and a 5 score. Mother–infant dyadic distance was aggregated over 2 weeks during the final LFD phase of the 16-week VFD cycle. The data were compared to two corresponding weeks of LFD in the non-VFD control group.

#### Blood and CSF Sampling

At the termination of the final LFD phase, all mothers from the VFD-exposure group were taken from their pens and placed in transfer cages in order to obtain single blood samples for plasma cortisol and cisternal CSF samples for OT. Subjects were released into a squeeze cage and intramuscular ketamine of 15 mg/kg was administered. Sedation was achieved in <5 min after returning to their respective transfer cages. Blood samples containing plasma cortisol were then rapidly obtained via venipuncture from the femoral vein preventing any potential rise in cortisol levels. Plasma cortisol was always performed between 9 and 11 a.m., to control for diurnal variation. Cisternal CSF samples were also obtained (4). Once anesthetized, 1.5 ml of CSF was withdrawn from the cisterna magna at a point directly below the occiput, as described by Scharf et al. (41). CSF samples were then placed in Gant tubes and stored in a −70°C freezer. Blood samples were deep freezed and later centrifuged and analyzed by radioimmunoassay procedures for plasma cortisol concentrations (18). CSF samples were analyzed for OT concentrations (30). Maternal non-VFD-exposed blood and CSF samples were obtained at an equivalent postpartum duration to the VFD-exposed mothers.

### Oxytocin analysis

Oxytocin standard was obtained from Peninsula Laboratories (Belmont, CA, USA). OT antiserum (kindly provided by D.A. Fisher) was diluted 1:5000. The OT antibody was raised in rabbit against synthetic OT peptide (not neurophysin). The standard curve concentration was 1–250 pg/ml. I^125^-OT tracer was obtained from New England Nuclear. The recovery of OT was over 95%. The sensitivity (90% binding) with serum concentrated twofold in the extraction was 2 pg/ml. All samples were run in one assay to eliminate interassay variability. The intra-assay coefficient of variation at 30 pg/ml was 6.2%.

### Statistical analyses

We inspected data for outliers and normality of distribution. Using *t*-tests, we compared infant age and weight and maternal age and weight to assess the need for covariates. Chi-square (2 × 2 tables) was performed to assess sex distribution of infants in the unstressed and VFD-exposed groups. Infant sex effects on their mothers were also ruled out by independent *t*-tests of relevant-dependent variables using infant sex as the independent variable. To test the influence of social rank on dyadic distance (“rank relevance”), a general linear model (GLM) utilized a factorial design. VFD exposure versus non-exposure served as the independent categorical measure, dyadic distance as the dependent measure and social rank (one = most dominant) as a continuous predictor variable. The interactive term of VFD exposure × social rank was also entered. The GLM was followed by *post hoc* Pearson’s correlations. A significant interactive effect would conceivably provide support for the hypothesis that maternal social rank was preferentially relevant to dyadic attachment patterns (significantly correlated) under stressful versus non-stressful maternal conditions. To validate the results, a similar GLM utilized social rank as a dichotomized categorical variable (“hierarchical status” was divided into subordinate versus dominant subjects), using a median split, rather than a continuous social ranking. The next GLM included plasma cortisol concentration as the dependent measure, VFD exposure versus non-exposure and dichotomized social rank (hierarchical status) as categorical variables, and VFD exposure × hierarchical status as the interactive variable. *Post hoct*-tests were applied. A significant interactive would conceivably provide support for the hypothesis that maternal stress resulted in relatively high cortisol in dominant versus subordinate mothers. We then examined, in a GLM, the effect of maternal body mass as a continuous predictor variable on maternal cortisol as a function of VFD exposure status. The interactive term entered was body mass × VFD exposure. A significant interactive effect would support the view that body mass predicted cortisol differentially as a function of maternal stress exposure. *Post hoc* Pearson’s correlations were performed. To delineate the relationship between body mass and maternal social rank in the current study, we divided mothers by median split into “high” versus “low” body weight to be used as the categorical variable in a GLM. The dependent variable was social rank. We hypothesized that increased maternal body mass was associated with increased dominance on social ranking. To assess the influence of maternal stress on the modulatory role of OT on the HPA-axis, we included OT as the continuous predictor variable and cortisol as the dependent variable with VFD exposure as the categorical variable. Should the interactive term of VFD exposure × OT be significant, it would conceivably provide support for the hypothesis that OT regulation of cortisol was induced by maternal VFD stress exposure. A follow-up OT/cortisol analysis was performed to further examine the predictive relationship of cortisol to OT under VFD exposure conditions. We dichotomized, using a median split, maternal plasma cortisol into “high” versus “low” plasma levels. Using a factorial design, a GLM then examined the effects of two categorical variables – VFD exposure and cortisol grouping, and their interactive effect – on OT. *Post hoct*-tests were performed. To examine the impact of hierarchical status on OT, we performed GLM analyses but with OT as the dependent variable and without cortisol grouping as a categorical variable.

Alpha level was set at *p* ≤ 0.05. All results were two tailed. Partial η^2^ was used to determine effect size where 0.14 or greater designated a large effect size (42).

## Results

No outliers were noted and all variables were normally distributed.

### Group comparisons

On *t*-tests, study subjects exhibited a mean (SD) infant age of 229 (39) days for VFD exposure; for the non-VFD-exposed infants the mean (SD) age was 201 (22) days [*t*-value = −1.97, df = 20, *p* = 0.062]. Because of the marginal differences between groups for infant age, we examined the relationship of infant age to variables of interest. There was no relationship between infant age and maternal–infant distance, maternal social rank, maternal plasma cortisol, or maternal CSF OT (*p* > 0.6 for all correlations). Infant age was, therefore, not routinely used as a covariate but key significant effects were tested with infant age added as a covariate to the model to exclude a confounding effect of infant age. Infant mean weight (SD) for VFD was 1.31 (0.27) kg; for non-VFD – 1.35 (0.24) kg [*t*-value = −0.33, df = 20, *p* = 0.73]. Mothers’ mean age (SD) in days for VFD = 2984 (1013); for non-VFD – 3142 (1522) [*t*-value = 0.29, df = 21, *p* = 0.77]. Mother’s mean weight for VFD = 5.23 (1.00) kg; non-VFD mean (SD) = 4.77 (0.93) kg [*t*-value = −1.14, df = 21, *p* = 0.26]. For sex distribution, of the 12 VFD infants, 7 were males and of the 11 non-VFD infants, 2 were male (Yates-corrected Chi-square = 2.38; df = 1; *p* = 0.12). The Yates correction was applied since one cell had less than five subjects. There were no infant sex effects on maternal social rank, maternal–infant dyadic distance, maternal plasma cortisol, and maternal CSF OT (all *p* > 0.13). Data on one infant age (non-VFD) and one infant weight (VFD) were not available.

### Relationship of social rank and dyadic distance

**Figure 1:**
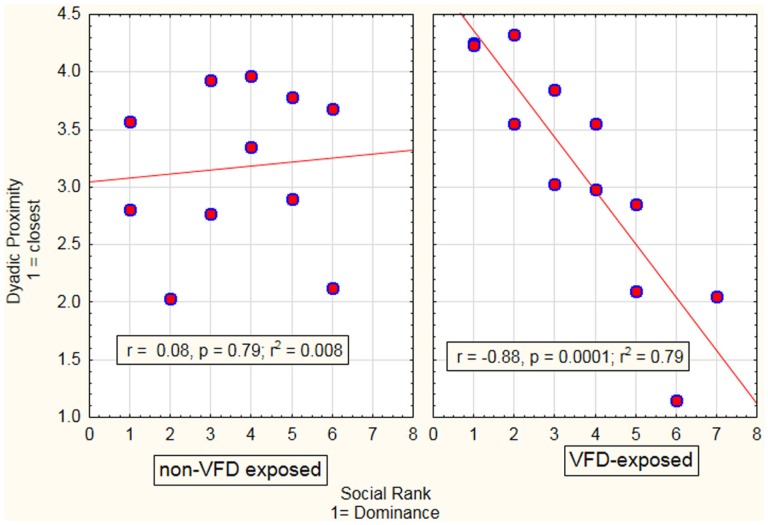
**Relationship between maternal–infant proximity as a function of maternal social rank in VFD-exposed versus non-exposed dyads**. A general linear model was performed with VFD exposure as the categorical variable, maternal social rank as the predictor variable and maternal–infant dyadic distance as the dependent variable. A significant interactive term of VFD exposure × maternal social rank [*F*_(1; 19)_ = 11.97; *p* = 0.003] indicated that predictive effects of social rank on dyadic proximity were specific to VFD mothers with low-social rank predicting decreased dyadic distance (see figure for respective Pearson’s correlations).

In the first GLM, we analyzed the effect of social rank as a continuous predictor variable on dyadic distance as a function of maternal stress exposure through assessment of the interactive term (see Figure 1). There was a social rank effect: mothers with relatively high-social rank exhibited increased dyadic-distance relative to subordinate mothers, who exhibited relatively decreased dyadic distance [*F*_(1; 19)_ = 8.87; *p* = 0.008]. There was a VFD exposure effect whereby reductions in dyadic distance in the VFD-exposed group were observed in comparison to the non-VFD-exposed group [*F*_(1; 19)_ = 9.35; *p* = 0.006]. A marked social rank by VFD exposure interaction was noted [*F*_(1; 19)_ = 11.97; *p* = 0.003; effect size: partial η^2^ = 0.39]. *Post hoc* Pearson’s correlation revealed that high-social rank predicted greater dyadic distance, and vice-versa, in VFD (*r* = 0.89, *N* = 12, *p* < 0.001; *r*^2^ = 79%) and was significantly distinguishable from non-VFD-exposed dyads (Figure 1), where no correlation (*r* = 0.09, *N* = 11, *p* = 0.79) between social rank and dyadic distance was observed. Effects remained significant when covaried for infant age.

When social rank was converted from a continuous to a dichotomous categorical variable using a median split, “hierarchical status” designating a dominant versus subordinate subgroup, we again noted a VFD exposure × hierarchical status interaction [*F*_(1; 19)_ = 8.32; *p* = 0.009]. Dyads with dominant mothers were significantly more distant from each other than subordinate dyads under the VFD exposure condition [Mean (SD) dyadic-distance score: dominant dyads = 3.87 (0.50), subordinate mean (SD) = 2.44 (0.85); *t*-value = 3.52, df = 11, *p* < 0.005)]. Under non-exposure conditions, there was no significant difference in dyadic-distance [mean (SD) dyadic-distance score: dominant dyads = 3.02 (0.74); subordinate dyads mean (SD) = 3.30 (0.68), *t*-value = −0.65; df = 10, *p* = 0.53].

### Relationship of hierarchical status to plasma cortisol and role of VFD exposure

In the next GLM, maternal plasma cortisol was used as the dependent variable, and categorical variables included VFD exposure versus non-exposure and hierarchical status (dominant versus subordinate). Using a factorial design, we entered the interactive term of VFD exposure × hierarchical status. There was a significant hierarchical status × VFD exposure effect in the prediction of maternal cortisol concentrations [*F*_(1; 19)_ = 5.52; *p* = 0.030]. We did not observe significant effects for VFD exposure or hierarchical status for basal maternal plasma cortisol. On *post hoct*-tests analysis (Table 1), in VFD-exposed mothers we observed relatively increased plasma cortisol level in dominant versus subordinate mothers (Table 1). In contrast, there were no differences observed for plasma cortisol as a function of hierarchical status in the non-VFD-exposed group (Table 1).

**Table 1:** **Effect of hierarchical status and VFD exposure on basal maternal plasma cortisol**.

	VFD-exposed mothers		Non-VFD-exposed mothers	
	
Rank	Dominant (*N* = 6)	Subordinate (*N* = 6)	Dominant (*N* = 6)	Subordinate (*N* = 5)
Mat. plasma cortisol conc. (μg/dl)	49.08 ± 3.61^a^	40.43 ± 6.28^a^	42.42 ± 2.34^b^	44.30 ± 7.22^b^

A general linear model was performed with two categorical variables, maternal VFD versus non-VFD exposure, and dominant versus subordinate groups (hierarchical status grouping), with plasma cortisol as the dependent measure. There was a significant VFD exposure × hierarchical status interactive effect for plasma cortisol [*F*_(1; 19)_ = 5.52; *p* = 0.03]. Dominant VFD-exposed mothers exhibited plasma cortisol elevations in comparison to subordinate VFD-exposed mothers ^a^whereas plasma cortisol in non-VFD-exposed mothers did not differ as a function of social rank^b^.

Mat., maternal; conc., concentrations.

*^a^*t*-Value = −2.92, df = 10, *p* = 0.015*.

*^b^*t*-Value = 0.55, df = 9, *p* = 0.59*.

### Relationship of maternal body mass to maternal plasma cortisol and role of maternal VFD exposure

In the next GLM, we examined the predictive effect of maternal body mass on maternal plasma cortisol level as the dependent variable and VFD exposure was used as a categorical variable. The interactive term of VFD exposure × maternal body mass was entered. There was a general effect of increased cortisol level in the VFD-exposed group in comparison to the non-VFD-exposed group [*F*_(1; 19)_ = 27.72; *p* = 0.001], when adjusted for body mass. A marked maternal weight by VFD exposure interaction was noted [*F*_(1; 19)_ = 29.53; *p* = 0.0001; partial η^2^ = 0.60]. On *post hoc* Pearson’s correlations, a positive correlation between maternal weight and maternal cortisol level for the VFD-exposed group was observed (*r* = 0.85, *N* = 12, *p* = 0.001) versus a negative correlation in the non-VFD group (*r* = −0.67, *N* = 11, *p* = 0.02). Thus, VFD exposure altered the relationship between maternal plasma cortisol levels to body mass from a negative to a positive relationship. Maternal body mass, when divided into a “heavy” versus “light” grouping by median split, significantly predicted social rank in the expected direction [*F*_(1; 19)_ = 6.88; *p* = 0.017]. “Heavy” mothers were dominant versus “light” mothers [“heavy” mothers mean (SD) = 2.75 (1.60) *N* = 12 versus “light” mothers mean (SD) = 4.55 (1.63) *N* = 11; *t*-value = 2.66; df = 21, *p* = 0.015]. A low score for social rank designates dominance. Thus, body mass was positively related to social rank.

### Relationship of CSF ot to plasma cortisol and influence of VFD exposure

**Figure 2:**
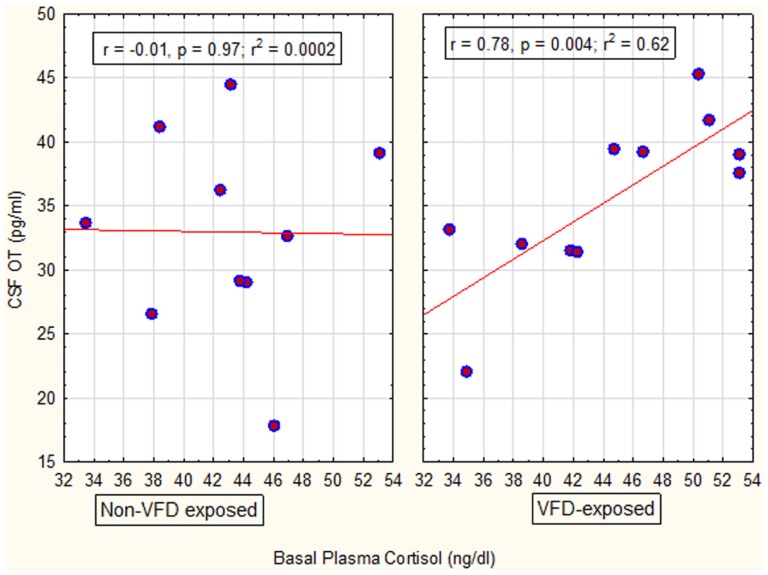
**Relationship between maternal CSF OT concentrations to maternal basal cortisol concentrations as a function of VFD exposure**. There was an overall VFD exposure effect with higher maternal plasma cortisol following VFD exposure versus non-exposure, when adjusted for maternal CSF OT [*F*_(1; 17)_ = 6.03, *p* = 0.025]. There was an overall OT effect positively predicting maternal cortisol [*F*_(1; 17)_ = 6.28, *p* = 0.022]. However, there was a significant VFD exposure × maternal CSF OT interactive effect [*F*_(1; 17)_ = 6.56, *p* = 0.02] with OT directly predicting cortisol in VFD-exposed mothers and an absence of relationship in non-VFD-exposed mothers (see figure for respective Pearson’s correlations).

In the next GLM, we used maternal plasma cortisol as the dependent variable, VFD exposure as the categorical variable and maternal CSF OT as the independent predictor variable. Consistent with a factorial design, we entered the interactive term of VFD exposure × maternal CSF OT into the GLM. There was an overall VFD exposure effect with higher maternal plasma cortisol following VFD exposure versus non-exposure when adjusted for maternal CSF OT [VFD-exposed mean (SE) = 44.60 (1.55) versus non-VFD-exposed mean (SE) = 42.97 (1.63); *F*_(1; 17)_ = 6.03, *p* = 0.025]. There was an overall OT effect positively predicting maternal cortisol [*F*_(1; 17)_ = 6.28, *p* = 0.022] and a significant VFD exposure × maternal CSF OT interactive effect [*F*_(1; 17)_ = 6.56, *p* = 0.02]. *Post hoc* Pearson’s correlations revealed a significant positive correlation in VFD-exposed mothers between maternal CSF OT and maternal plasma cortisol (*r* = 0.78; *N* = 11, *p* = 0.004), whereas the corresponding correlation in non-exposed mothers was absent (*r* = −0.01; *N* = 10, *p* = 0.97) (Figure 2). Thus, OT was positively predictive of cortisol although this effect was confined to the VFD-exposed mothers. Two OT values were not available for analysis. Effects remained significant when covarying for infant age.

**Figure 3:**
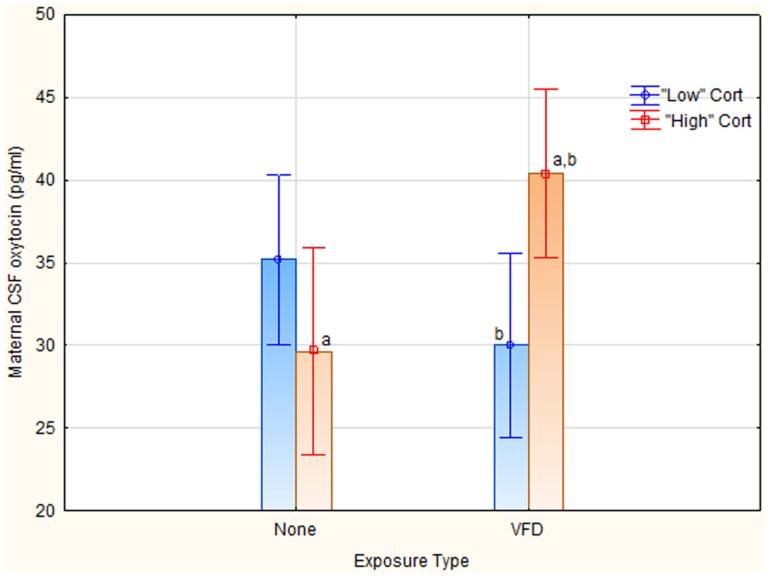
**Relationship between maternal plasma cortisol grouping to maternal CSF OT concentrations as a function of VFD exposure**. We dichotomized, using a median split, maternal plasma cortisol into “high” versus “low” plasma levels [“high” cortisol mean (SD) = 48.96 (3.57) ng/ml (*N* = 10) versus “low” cortisol mean (SD) = 39.16 (3.84) ng/ml (*N* = 11); *t*-value = −6.02;df = 19; *p* = 0.000008]. Two cortisol values lacking matching OT values were excluded. Using a factorial ANOVA GLM with OT as the dependent variable, and cortisol grouping and VFD exposure as categorical variables, there was no VFD exposure effect, or cortisol grouping effect but a VFD exposure × cortisol grouping interaction was noted [*F*_(1; 17)_ = 9.23, *p* = 0.007]. *Post hoct*-test differences (*p* ≤ 0.05) for CSF OT; ^a^VFD-exposure high-cortisol grouping > non-exposed high-cortisol grouping; ^b^VFD-exposure high-cortisol grouping > VFD-exposure low-cortisol grouping. Vertical bars denote 0.95 confidence intervals.

A second question was whether maternal plasma cortisol was predictive of maternal CSF OT. However, maternal plasma cortisol as a continuous predictor variable did not predict maternal CSF OT, even in an interactive fashion. A second OT/cortisol non-parametric analysis was therefore performed to examine the predictive relationship of cortisol to OT under VFD exposure conditions. We dichotomized, using a median split, maternal plasma cortisol into “high” versus “low” plasma levels [“high” cortisol mean (SD) = 48.96 (3.57) ng/ml (*N* = 10) versus “low” cortisol mean (SD) = 39.16 (3.84) ng/ml (*N* = 11); *t*-value = −6.02; df = 19; *p* = 0.000008]. Of note, there were no VFD exposure effects on maternal plasma cortisol [VFD-exposure mean (SD) = 44.60 (6.95) ng/ml (*N* = 11) versus non-VFD-exposed mean (SD) = 42.97 (5.45); *t*-value = −0.59; df = 19; *p* = 0.55], which contrasts to the cortisol effect when controlling for CSF OT concentrations and body mass. Two cortisol value lacking matching OT values were excluded. Using a factorial ANOVA GLM with OT as the dependent variable, and cortisol grouping and VFD exposure as categorical variables, there was no VFD exposure effect or cortisol grouping effect but a VFD exposure × cortisol grouping interaction was noted [*F*_(1; 17)_ = 9.23, *p* = 0.007] (see Figure 3). Effects remained significant when covarying for infant age. High “cortisol” subjects that were VFD-exposed [mean (SD) = 40.36 (2.73) (*N* = 6)] exhibited elevated maternal CSF OT (pg/ml) compared to low “cortisol” VFD subjects [mean (SD) = 29.98 (4.52) (*N* = 5); *t*-value = −4.70; df = 9, *p* = 0.001]. The same comparison was not significant in non-exposed mothers. VFD-exposed mothers from the high cortisol grouping also exhibited significantly greater CSF OT than non-exposed mothers with high cortisol [mean (SD) = 29.63 (8.96) *N* = 4; *t*-value = −2.81, df = 8, *p* = 0.02].

We then again validated that VFD-exposed mothers with “high” cortisol displayed significantly higher social rank than those with “low” cortisol [1 = most dominant; high-cortisol mean (SD) = 2.43 (1.40); *N* = 6, compared to low-cortisol mean (SD) = 5.20 (1.30); *N* = 5, *t*-value = 3.48; df = 9; *p* = 0.006]. The corresponding comparison in non-VFD-exposed mothers was not significant. Thus, although there was no VFD-exposure effect or cortisol grouping effect on social rank, there was a significant VFD exposure × cortisol grouping interaction [*F*_(1; 17)_ = 7.10; *p* = 0.02]. Thus, VFD exposure in conjunction with high cortisol exhibited both elevated OT and was associated with higher social rank compared to VFD-exposed mothers with low cortisol. In non-VFD-exposed mothers, cortisol grouping did not predict either CSF OT or social rank [Cortisol grouping × VFD exposure interaction using both social rank and CSF OT as dependent variables: Wilks λ = 0.51; df = 2.15; *p* = 0.005].

### The relationship of OT to hierarchical status and potential influence of VFD exposure

**Figure 4:**
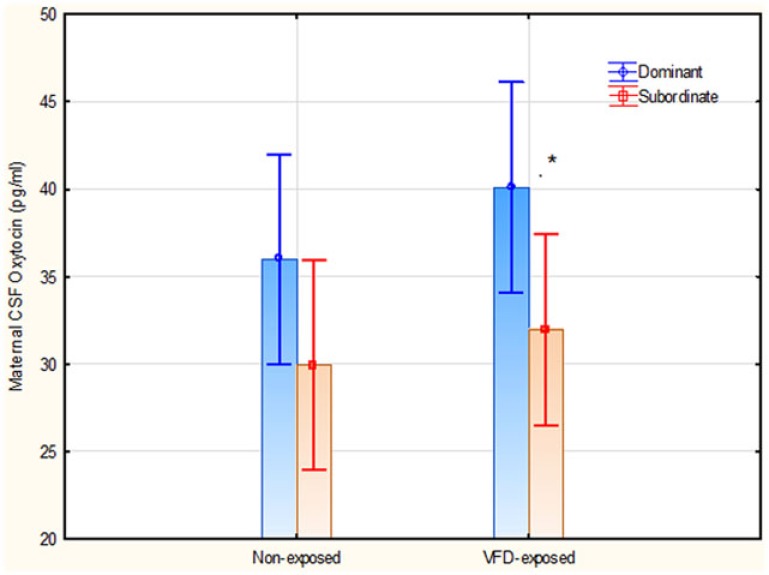
**Maternal CSF OT concentrations as a function of VFD exposure and hierarchical status and their interaction**. There was an overall effect of hierarchical status, with dominant mothers exhibiting greater CSF OT concentrations than subordinate mothers. [*F*_(1; 17)_ = 6.45; *p* = 0.021]. *Post hoc testing revealed that dominant VFD-exposed mothers exhibited significantly greater CSF OT than subordinate VFD-exposed mothers. No interactive effects were noted. Vertical bars denote 0.95 confidence intervals.

The next GLM aimed to determine the effects of VFD exposure and hierarchical status on maternal CSF OT concentrations independent of cortisol levels. The interactive term of VFD exposure × hierarchical status was entered into a factorial design. There was an overall effect of hierarchical status, with dominant mothers exhibiting greater CSF OT concentrations than subordinate mothers. [Dominants mean (SE) = 38.04 (2.02), *N* = 10 versus Subordinates mean (SE) = 30.94 (1.93), *N* = 11; *F*_(1; 17)_ = 6.45; *p* = 0.021] (Figure 4). There was no effect of VFD exposure on maternal CSF OT levels and the VFD exposure × hierarchical status interaction was not significant. *Post hoct*-tests revealed significantly greater maternal CSF OT in dominants versus subordinates in VFD-exposed mothers [Dominant mean (SD) = 40.09 (2.97), *N* = 5 versus subordinate mean (SD) = 31.94 (6.27), *N* = 6, *t*-value = 2.65; df = 9, *p* = 0.026], whereas in non-VFD, there were no hierarchical status differences for maternal CSF OT. Two maternal CSF OT values were not available for analysis.

## Discussion

Early-life stress is thought to be an important mediator of various psychiatric illnesses including depressive (1, 43), anxiety (43), substance use (44), and pain disorders (45). VFD is an established animal model of early-life stress where manipulating maternal accessibility to food, without nutritional deprivation, creates an unpredictable and inconsistent rearing environment for infant non-human primates. The repeated shifting of foraging demand appears to overwhelm maternal coping capacities and induce a form of “functional” emotional separation between mother and infant (8). The psychosocial stress induced by the VFD paradigm has a putative impact on the ability of primate mothers to provide consistent nurturing toward their infants (8).

**Figure 5:**
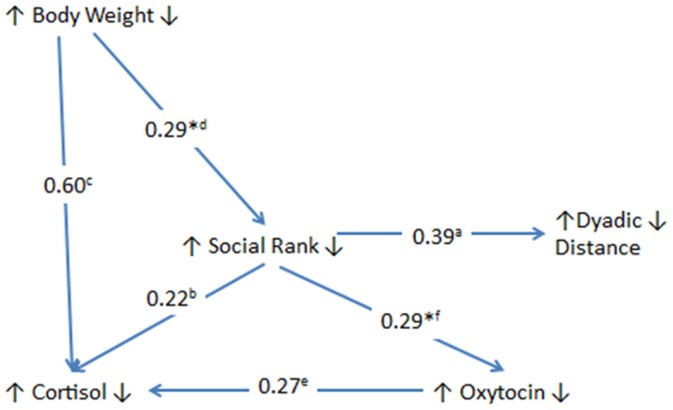
**Relationship and effect sizes among maternal variables exposed to variable foraging demand versus unstressed control mothers**. The figure schematizes the effect of VFD exposure versus controls as described by the effect size of the interactive effect of VFD exposure × predictor variable (line inception) in the prediction of the dependent variable (arrow point) except for those effect sizes denoted by an asterisk (where no group effects were evident). Directional arrows in front of each variable are all in an upward direction and at the rear of each variable are all in a downward direction indicating a direct relationship for all variables studied in VFD-exposed mothers versus control mothers (except those relationships that are designated by an asterisk). a = social rank by VFD exposure interaction predicting dyadic distance [*F*_(1; 19)_ = 11.97; *p* = 0.003]. b = hierarchical status by VFD exposure effect in the prediction of maternal cortisol concentrations [*F*_(1; 19)_ = 5.52; *p* = 0.030] (dominants exhibited higher cortisol than subordinates). c = maternal weight by VFD exposure interaction in the prediction of maternal cortisol concentrations [*F*_(1; 19)_ = 29.53; *p* = 0.0001]. *d = greater body weight predicted greater maternal dominance [*F*_(1; 19)_ = 6.88; *p* = 0.017] without interactive effect. e = VFD exposure × maternal CSF OT interactive effect in the prediction of maternal cortisol concentrations [*F*_(1; 17)_ = 6.56, *p* = 0.02]. *f = dominant mothers exhibited greater CSF OT concentrations than subordinate mothers [*F*_(1; 17)_ = 6.45; *p* = 0.021] without interactive effect. Partial η^2^: large effect size ≥ 0.14. Note, except for (d) and (f), all other correlational relationships are significant in VFD-exposed but not non-exposed mothers.

In our attempt to decipher important factors in the transmission of VFD-exposure stress from mothers to their infants, we discovered a complex interaction among maternal social rank, dyadic distance, maternal plasma cortisol, and maternal CSF OT (see Figure 5) with the most salient finding of the current study highlighting the pivotal role of maternal social rank, particularly under VFD-exposure conditions. Clearly, social rank plays a disproportionate role in the context of a perceived shortage of food resources engendered by VFD exposure. Either as a continuous variable, social rank, or as a dichotomous variable, “hierarchical status,” (dominant versus subordinate), the variable significantly predicted among the VFD group, (1) maternal dyadic distance between mother and infant (high rank = increased dyadic distance), (2) maternal plasma cortisol (dominance = high cortisol), and (3) CSF OT directly predicts plasma cortisol but specifically in VFD exposure (upon VFD exposure, high OT predicts high cortisol). Among both VFD and non-VFD groups, social rank predicts CSF OT levels (dominance = increased CSF OT).

However, ultimately social rank is significantly predicted by body mass independent of VFD exposure, suggesting that the final predictor of all our variables is predicated by the balance of access to, and requirement of, food and that this factor, vital for survival of mother and infant, is rendered of utmost relevance upon VFD exposure. Partial η^2^ effect sizes for the above relationships are all well in the large range (Figure 5) ranging from 0.22 to 0.60, where 0.14 designates a large effect size. All key results remain significant when covarying for infant age, a potentially confounding between-group variable. Moreover, infant sex, although not differentially distributed between groups, does not predict any of the key maternal variables. The study underlines the substantial yet divergent plasticity that is evident in maternal physiology and behavior, depending on dominant versus subordinate status, with persistent sequelae evident in VFD offspring, ostensibly independent of maternal social rank (40).

We therefore postulate that the socioecological instability engendered by maternal VFD exposure heightens the relevance of social rank, and this heightened relevance is reflected by social rank strongly predicting dyadic distance in the VFD group. The positive association we found between social rank and dyadic distance under VFD conditions is consistent with previous studies of Blue Monkeys, where the authors analyzed maternal protection of their infants via restriction of their movements. Maternal restrictiveness appeared to be related to social rank: low ranking mothers increased restrictiveness in order to, presumably, ensure access to their infants while high-ranking mothers reduced restrictiveness in order to, according to the authors, enhance their foraging efficiency (28). Offspring of high-ranking monkeys may be in less need of constant protection from other adult females. Relating their findings to the socially housed bonnet macaques in the current study, we would expect the relationship between social rank and dyadic distance or “maternal restrictiveness” to become more pronounced under VFD conditions, where foraging demands are considerably greater in comparison to non-VFD conditions.

Further buttressing the inordinate influence social rank plays under conditions of socioecological instability, dominant and subordinate mothers exhibit divergent patterns of HPA-axis regulation, the former exhibiting greater cortisol than the latter. Non-exposed macaque mothers exhibit intermediate plasma cortisol levels that are not distinguishable as a function of hierarchical status. In an additional study in our laboratory, and not apparent in this smaller number of subjects, non-VFD-exposed dominant mothers exhibited relatively low cortisol levels, while subordinate animals exhibited relatively higher cortisol levels (40), suggesting that maternal VFD exposure reverses the relationship of hierarchical status to HPA-axis activity. The divergence may conceivably arise as VFD dominants may be more active and VFD subordinates less active since they are staying near their infants (46). Non-VFD dominants may be less stressed compared to non-VFD subordinates accounting for the opposite cortisol relationship.

However, our hierarchical status and maternal plasma cortisol findings are consistent, in part, with those of a study in human males, which assessed the effects of sustained stress (boot camp training) and social rank on adrenocortical activity and reactivity (25). Under experimental psychological and physical stress, dominant and subordinate subjects exhibited different patterns of cortisol response, with socially dominant men exhibiting markedly increased salivary cortisol levels throughout the training period and subordinate men exhibiting only modest increases.

Finally, we studied the relationship between central levels of OT and social rank and OT and plasma cortisol under VFD exposure and non-exposed conditions. We found central OT levels to vary as a function of hierarchical status, irrespective of VFD exposure, with a positive association between dominance and central OT levels (Figure 4). This is consistent with findings of previous studies where peripheral OT was found to be higher in dominant versus subordinate rhesus macaques. The authors speculate OT may facilitate dominance either because dominant monkeys are more likely to initiate affiliative behaviors under the influence of OT (32), or OT may blunt social vigilance toward social threats (34).

With regards to HPA-axis regulation, we found positive correlation between central OT concentrations and plasma cortisol specifically in the VFD-exposure condition (Figure 3). This relationship was observed with central OT as a predictor variable, and not under non-VFD conditions. We speculate that under conditions of maternal VFD exposure, OT, consistent with its HPA-axis suppression effects (37), restrains HPA-axis activation in a compensatory manner when socioecological instability is prolonged. This may be viewed as an adaptation to allostatic overload (47). Thus, high OT may restrain further HPA-axis activation in dominant VFD mothers who have high-cortisol levels (Figure 4), possibly due to high-physical activity, whereas the low-cortisol levels evident in VFD-exposed subordinates does not necessitate OT elevations.

The clinical relevance of the current study exists at several levels. Childhood maltreatment and/or neglect is a major precursor for a variety of human adult disorders including mood (48–51) and anxiety disorders (49, 52), metabolic syndrome (53), chronic pain disorders (51, 54, 55), and cardiovascular disease (50, 56, 57). The impact of childhood adversity on the central nervous system is therefore particularly critical to the pathophysiology of certain anxiety and mood disorders. Yet, interventions buttressing maternal vulnerability, which would protect against negative sequelae in their offspring (58), would be facilitated by cogent animal models in non-human primates. OT, another dependent variable in the study, has been implicated in psychiatric disorders, such as autism (59), schizophrenia (60), and depression (61). Exogenous administration of OT promotes pro-social behavior and may improve social function (34). Therefore, further understanding of the role of OT in buttressing allostatic overload (62) and in disorders of social function are important.

Limitations of the study include a non-significant yet relative imbalance of infant sex between the groups, although infant sex had no bearing on maternal variables of relevance. Infant age was also marginally greater in VFD-exposed dyads than non-stressed controls, but, again, infant age had no bearing of variables of interest and results remained significant when covaried for infant age. Plasma cortisol, a marker of HPA-axis activity, was only drawn on a single occasion. More detailed assessment of the HPA-axis may be warranted. Yet, the single cortisol sample was sufficient to demonstrate significant effects that were in accordance with the study’s hypotheses. Although preferable to plasma OT as a reflection of central OT, cisternal CSF OT provides little information regarding regional OT concentrations and OT receptor changes. Such information would be feasible only through terminal studies, outweighed not least by the need for mothers to care for their infants. Vasopressin is also invoked as an important component of the central OT system (63, 64) and measurement of vasopressin would enhance the value of the study and understanding of OT physiology. Dyadic distance alone may not adequately reflect the full repertoire of maternal and infant behavioral responses (27). Future studies should include more comprehensive behavioral analyses. Yet, the effect size of the difference in correlation between social rank and dyadic distance in VFD-exposed compared to non-VFD dyads (almost 80% of the variance in VFD-exposed dyads) was strong, suggesting that the measure was valid with regard to its negative impact on maternal contingent responsivity.

Our findings suggest that under conditions of socioecological stress, social rank becomes increasingly relevant as reflected by both dyadic distance between mother and infant and HPA-axis regulation. While CSF OT did reflect the relevance of social rank – dominant mothers exhibited increased levels of CSF OT in both VFD and non-VFD conditions – its regulation of HPA-axis specifically under VFD-exposure conditions implies participation of OT effects on HPA-axis under conditions of socioecological stress. These findings would appear important in demonstrating divergent maternal plasticity in response to stress as a function of social rank. Divergent maternal physiological and behavioral responses appear, therefore, adaptively imperative, yet both, we argue, compromise the mother’s ability to provide adequate nurturance to her developing infant because of disproportionate attention to maternal rank. Predictability seems to be an important factor in stress response in a social group (65). Socioecological instability, therefore, constitutes a major stressor for mothers, requiring widespread maternal adaptations, and the diversion of maternal capacity ideally conserved for contingent responsivity to her infant in the service of balancing foraging needs, child rearing and social rank within the social group (40).

## Conflict of Interest Statement

The authors declare that the research was conducted in the absence of any commercial or financial relationships that could be construed as a potential conflict of interest.
